# Tissue P16 is Associated with Smoking Status among Indonesian Nasopharyngeal Carcinoma Subjects

**DOI:** 10.31557/APJCP.2019.20.7.2125

**Published:** 2019

**Authors:** Laila Wahyuningsih, Ery Kus Dwianingsih, Erika Diana Risanti, Prijono Tirtoprodjo, Hanggoro Tri Rinonce, Fikar Arsyad Hakim, Camelia Herdini, Jajah Fachiroh

**Affiliations:** 1 *Department of Anatomical Pathology, Faculty of Medicine, Public Health and Nursing,*; 3 *Department of Otorhinolaryngology Head and Neck Surgery, *; 4 *Department of Histology and Cell Biology, Universitas Gadjah Mada (FK-KMK UGM), Yogyakarta, *; 2 *Faculty of Medicine, Universitas Muhammadiyah, Surakarta, Indonesia. *

**Keywords:** P16- nasopharyngeal carcinoma- smoking status- Indonesia

## Abstract

**Background:**

Nasopharyngeal carcinoma (NPC) is a malignancy with high incidence in Southern China and South-East Asia. NPC incidence among males in Indonesia is estimated around 8.3/100,000 populations. Tobacco smoking is a common risk factor for cancer, including NPC. P16 is one of the key proteins related to the activation of apoptotic pathways, that commonly change during carcinogenesis. Carcinogenesis is often related to environmental exposure, including tobacco smoke.

**Objective:**

To analyze the association between P16 protein and smoking status among NPC subjects in Indonesia.

**Methods:**

Forty formalin fixed-paraffin embedded NPC tissue samples of known smoking status (20 smokers, 20 non-smokers) were collected from the Department of Anatomical Pathology, Dr. Sardjito Hospital, Yogyakarta. P16 was detected by immunohistochemistry staining. German semi-quantitative scoring system was applied to the P16 staining. Expression index with the score of 0 to 3 was classified as negative staining, meanwhile 4 to 12 was classified as positive staining. The association between P16 (score) and smoking status among NPC patients was analyzed using Fischer exact test. One-sided p ≤ 0.05 was considered as statistically significant.

**Results:**

All samples were Javanese males, with age range 25-76 years old. P16 positive staining among smokers was 5% (1/20), while among non-smokers was 40% (8/20). P16 among smokers was significantly lower than non-smokers patients (p=0.010). No difference was found between quantity of smoke and P16 score.

**Conclusion:**

A significant association between P16 and smoking status in Indonesian NPC patients has been revealed. The result of this study may be used to improve prevention and management of NPC cases related to smoking habit in Indonesia.

## Introduction

Nasopharyngeal carcinoma (NPC) is a malignant tumor that grows in the mucosal epithelium of the posterior nasopharyngeal wall. NPC is histopathologically divided into 3 subtypes: keratinizing squamous-cell carcinoma (type I), differentiated non-keratinizing carcinoma (type II) and undifferentiated non-keratinizing carcinoma (type III) (Barnes et al., 2005). Type I NPC is more common in non-endemic areas; whereas type II and III are more common in endemic areas, and often associated with Epstein-Barr virus (EBV) infection (Tulalamba and Janvilisri, 2012). 

P16 is tumor suppressor gene (TSG), a cyclin dependent kinase (CDK) inhibitor that regulates tumor cells through the G1 phase of the cell cycle that is often inactivated in cancer (Shao et al., 2014). Inactivation of P16 has been found in nearly 50% of all human cancers (Li et al., 2011). Previous NPC studies reported that P16 gene expression was reduced or absent in the majority of patients, which may result in the increase of cellular proliferation (Gulley et al., 1998; Makitie et al., 2003) Hypermethylation of P16 gene which causes P16 gene inactivation plays an important role in NPC carcinogenesis. Inactivation of P16 gene occurs during the early stages of the disease and this may be useful in the detection of early stage (Tam et al., 2013; Shao et al., 2014). Additionally, NPC cell line treated with P16 gene therapy to normalize its P16 protein level showed an improved postradiation outcome (Wang et al., 1999; Chou et al., 2008). 

NPC was estimated as the fifth most common cancer among Indonesian men. The incidence of NPC in Indonesia was estimated to be 8.3/100,000 population per year, and the mortality rate was 5/100,000 population per year (Ferlay et al., 2012). Indonesia has the third largest number of smokers in the world, after China and India (WHO, 2012). The proportion of male smokers >15 years old reached 64.9% in 2013 (Badan Penelitian dan Pengembangan kesehatan-Kementerian Kesehatan RI, 2013). Previous studies have shown that smokers are at higher risk of developing NPC than non-smokers (Fachiroh et al., 2012; Xue et al., 2013; Xie et al., 2015; Yong et al., 2017). The younger the smoking is started, the higher the risk of developing NPC (Hsu et al., 2009). 

This research is the first study reporting the analysis on the association of P16 gene expression (“P16 protein/ P16”) and smoking status among males with NPC in Indonesia. This report is expected to reinforce the understanding of the molecular mechanisms of NPC associated with patient risk factors for better prevention and management. 

## Materials and Methods

*Samples and data collection*

A hospital-based case control study on NPC was conducted from 2012 until 2016 (Principle investigator: Jajah Fachiroh). Demographic, clinical, and environmental exposure (dietary and non-dietary habits) information were collected through survey. A nested study using recruited data of NPC males with known smoking status was done. From these data, 40 formalin-fixed paraffin embedded (FFPE) tissues were collected (20 samples from tobacco smokers and 20 samples from never-smokers) from the Department of Pathological Anatomy, Dr. Sardjito General Hospital, Yogyakarta, Indonesia. Data on demographics, medical history and details of smoking habit were retrieved from the research database, including smoking duration and number of cigarettes smoked per day. For this study, tobacco smokers were determined as those who was smoking regularly for at least a year, while non-smokers were those who never smoked. All pathological specimens were reviewed and reclassified by expert pathologists to ensure tumor homogeneity, by having tumor cell count of at least 30%. 

The research was approved by The Medical and Health Research Ethics Committee (MHREC) of the Faculty of Public Health and Nursing Universitas Gadjah Mada (FK-KMK-UGM), Yogyakarta, Indonesia and has obtained a study sampling permit from Dr. Sardjito Hospital, Yogyakarta, Indonesia.

**Table 1:** Demographic and Clinicopathological Features of the Subjects

Characteristic	Smoker n (%)	Non-smokers (%)
Age		
≤50 years old	9 (45)	9 (45)
>50 years old	11 (55)	11 (55)
	51.03±10.57	
Mean	50.90±10.22	51.15±11.17
Range	25-76	
	33-76	25-66
WHO Type		
1	0 (0)	0 (0)
2	0 (0)	1 (5)
3	20 (100)	19 (95)
Tumor Stage		
2	1 (5%)	2 (10%)
3	2 (10%)	5 (25%)
4	15 (75%)	13 (65%)

**Table 2:** The Association between P16 Score and Smoking Status of NPC

Characteristic		Smokers n (%)	Non-smokers n (%)	P-value**
Staining extent in the tumor cells	0%=0	5 (25)	6 (30)	
	1-10%=1	9 (45)	4 (20)	
	11– 50% = 2	6 (30)	9 (45)	
	81–100% = 4	0 (0)	1 (5)	
Intensity of the nucleic stain	no stain = 0	5 (25)	6 (30)	
	weak stain = 1	13 (65)	9 (45)	
	moderate stain = 2	2 (10)	5 (25)	
	strong stain = 3	0 (0)	0 (0)	
Expression Index (EI)*	1-3 (negative)	19 (95)	12 (60)	0.01
	4-12 (positive)	1 (5)	8 (40)	

**Table 3:** The Association between Smoking Intensity and P16 Score in NPC

	P16 gene Expression Index (EI)		*P-value* *
Characteristic	1-3 (Negative)	4-12 (Positive)	
Smoking Duration (Year)			
≤30	8 (40%)	0 (0%)	1
>30	11 (55%)	1 (5%)	
Cigarette per day			
≤10	10 (50%)	0 (0%)	1
>10	9 (45%)	1 (5%)	
≤30	15 (75%)	0 (0%)	0.25
>30	4 (20%)	1 (5%)	

* Fisher’s exact test analysis, one sided p value ≤ 5 considered as statistically significant

**Figure 1:**
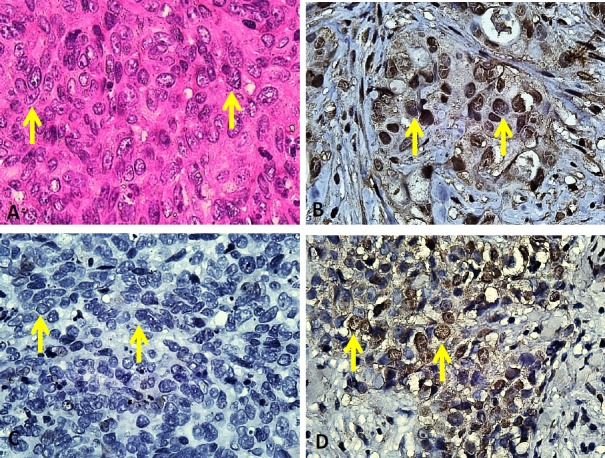
Histopathologic Feature of NPC and P16 Expression in NPC Patients and Pancreatic Carcinoma as a Positive Control. (A) Microscopic feature of NPC, showing infiltrative solid tumor nest (Hematoxylene Eosin (HE), 400X). (B) Nuclear staining of P16 expression in positive control (400X). (C) Negative expression of P16 in patient sample (400X). (D) Positive expression of P16 in patient sample (400X)

*Immunohistochemistry (IHC) staining*

FFPE samples were cut into 3µ thick slide for IHC staining. FFPE sections were incubated, deparaffinized, and rehydrated. Antigen retrieval was performed using a decloaking chamber (BioCare Medical, USA). Detection of P16 was done by using monoclonal antibody anti-CDKN2A/ P16INK4a clone 2C5 (LSBio, USA) diluted to 1:150 in phosphate buffer saline (PBS). Diamino-benzidine (DAB) for visualization of positive cells was applied with a semi-automatic Intellipath FLX (BioCare Medical, USA) according to the manufacturer’s instructions. Pancreatic carcinoma tissue was used for P16 staining positive control. 

*Interpretation of P16 IHC results*

P16 protein was observed under a light microscope by an independent and experienced pathologist. German semi-quantitative scoring system was used, by considering the staining intensity and staining extent (Kok et al., 2010). Each sample was given a score according to the intensity of the nucleic stain (no stain = 0, weak stain = 1, moderate stain = 2, strong stain = 3) and the area fraction stained in the tumor cells (0% = 0; 1–10% = 1; 11– 50% = 2; 51–80% = 3; 81–100% = 4). Area fraction score was assessed by counting nuclear positive stained per 500 tumor cells in 5 high power fields (Matos et al., 2006). The final immunoreactivity score (Expression Index) was determined by multiplying the three-tier intensity score with the four-tier area fraction score, with the minimum score of 0 and a maximum score of 12. The cut-off point was set at 4. Expression index 0-3 was classified as negative ( or “loss of P16 gene expression”), while 4-12 was classified positive (with at least moderate staining in 11-50% tumor cells) (Kok et al., 2010).

*Statistical analysis*

Pack year was calculated based on the number of cigarettes smoked per day divided by 20, and multiplied by the year of smoking. Differences of years of smoke, number of cigarettes smoked per day and pack years were based on the median of each variable among the smokers.

The association between P16 and smoking status (including years of smoking, number of cigarettes smoked per day, and pack years) were analyzed using Fisher’s exact test. A probability value of equal or less than 0.05 (1-sided) was considered as statistically significant.

## Results

*Demographic and clinicopathological features of NPC patients*

The demographic and clinicopathological features of the research subjects are summarized in Table 1. All subjects were male, Javanese ethnicity with age range from 25 to 76 years old (mean 51.03±10.57). Histopathological examination according to the World Health Organization (WHO) categories showed almost all subjects were WHO type 3 category, with one WHO type 2. All subjects were at late stage. 

*Immunohistochemistry (IHC) Analysis of P16*

Histopathologic features of NPC and P16 detection in the tissue samples are shown in Figure 1. Hematoxylin-eosin (HE) staining of the tissues showed microscopic features of NPC, characterized with solid and infiltrative tumor nests, polymorphic cells with round-oval shapes, spindle nuclear with coarse chromatin and visible nucleoli. The nuclear immunoreactivity of the P16 was considered as positive and further used for analysis. 

*P16 and Smoking Habit*

The analysis of P16 score among smoker and non-smoker groups is summarized in Table 2. The P16 examination showed the extent of the positive areas varied from 0-80% in both groups, while the intensity of the nucleic stain varied from no stain to moderate stain. 

In the smoker group, 19/20 (95%) showed P16 score below the cut-off level (expression index 1-3 or “loss of P16 gene expression”), while in the non-smoker group, 12/20 (60%) showed loss of P16 gene expression. Further analysis showed no correlation between P16 score with smoking intensity (Table 3). 

## Discussion

Nasopharyngeal carcinoma is a cancer with a unique distribution worldwide. The Southeast Asia region is a hotspot for NPC (Ferlay et al., 2012). The geographical and racial profiles play roles in the NPC incidence (Tulalamba and Janvilisri, 2012). NPC is more prevalent in males compared to females, and the incidence tended to increase with age (Ferlay et al., 2012; Nawaz, 2015a), while in Indonesia 17% -21% of NPC cases were diagnosed at ages less than 30 years old (Adham et al., 2012). NPC is sensitive to radiation therapy; however, NPC has a poor prognosis because of its hidden location and non-specific early symptoms, which accounts for more than 50% of cases diagnosed at an advanced stage with local expansion (Tulalamba and Janvilisri, 2012; Peng et al., 2014; Nawaz et al., 2015b) that requires more complicated treatments.

Indonesia is estimated to have an intermediate incidence of NPC, characterized by peak incidence at later age with majority of WHO type II/ III (Adham et al., 2012) On the other hand, NPC in non-endemic populations had double peaks of incidence at adolescence and late adulthood (Bray et al., 2008), with the majority with WHO type I. 

WHO data showed that Indonesia has the third largest number of adult tobacco smokers in the world, after China and India. The majority of active smokers in Indonesia are male, amounting to 47.5% while only 1.1% of women (Badan Penelitian dan Pengembangan kesehatan-Kementerian Kesehatan R, 2013). Tobacco smoking is a known risk for NPC, from low to high incidence populations (Turkoz et al., 2011; Fachiroh et al., 2012; Jia and Qin, 2012; Yong et al., 2017; Long et al., 2017); this pattern may be in concordance to the predominant NPC incidence among males, compared to females. Exposure to carcinogens early in life could have substantial impacts on the development of NPC. Cigarette carcinogens act as mutagenic and DNA damaging agents that initiate tumorigenesis in normal nasopharyngeal epithelial cells (Hsu et al., 2009; Gupta et al., 2017; Long et al., 2017). 

The cumulative effect of exposure to tobacco smoking to the risk of NPC is significant and tends to be a dose-dependent pattern. The risk of developing NPC rises as the number of cigarettes smoked is increased (intensity of cigarettes smoked per day and the amount of pack years) (Fachiroh et al., 2012; Xue et al., 2013; Gupta et al., 2017; Long et al., 2017). 

In this study, in the NPC tissue, the loss of P16 gene expression (“P16 protein”) was much higher among smokers compared to non-smokers. This finding proved the association P16 gene expression and smoking status in NPC patients independent of disease stage (p=0.692; data not shown). However, we could not derive any correlation between P16 scores with smoking intensity among NPC. 

The mechanism of P16 gene inactivation was often caused by deletion, methylation, or gene mutation causing an alteration of protein production (Lo et al., 1996), similar to those found in NPC and non-small cell lung carcinoma (NSCLC) (Gulley et al., 1998; Tam et al., 2013). Studies of NSCLC cell lines showed that homozygous deletions were the most common mechanism of inactivation of the P16 gene, followed by methylation and mutation (Tam et al., 2013). Multiple studies have examined that down-regulation of P16 gene expression and its association with promoter hypermethylation is common in NPC (Mäkitie et al., 2003; Hutajulu et al., 2011; Lee and Pausova, 2013; Shao et al., 2014; Nawaz, 2015a; Jiang et al., 2016). Cigarette smoke is a powerful environmental modifier for DNA methylation (Lee and Pausova, 2013). In addition, cigarette smoke triggers a chronic nasopharyngeal mucosal inflammation that is facilitated in a person infected with EBV, in which Latent Membrane Protein 1 (LMP1) may be involved in P16 gene methylation (Mäkitie et al., 2003; Chou et al., 2008; Adham et al., 2012). Research on the Chinese population showed that cigarettes were related to DNA methylation (Zhu et al., 2016). Tobacco smoking has also been shown to be associated with P16 gene hypermethylation in NSCLC (Zhang et al., 2011). A study of the North Indian population also showed that smoking speeded up the hypermethylation of P16 genes, thus disabling their expression, making the person more susceptible to the risk of lung and other solid carcinomas (Deep et al., 2012). However Tam et al. failed to find a relationship between smoking and method of inactivation of P16 gene expression in NSCLC cases (Tam et al., 2013). 

Air pollutants derived from vehicle fumes, factory smoke or burning residues have the potential to cause cancer, but the risk of malignancy is lower when compared to cigarette smoke (Loomis et al., 2013). Other air pollutants (carcinogens) that may affect DNA methylation include formaldehyde, which is widely used in household products such as adhesives, fungicides, germicides and disinfectants (Zhu et al., 2005). No data have linked the effect of formaldehyde to expressions of the P16 gene on NPC. The effect of pollutants (including secondary/ tertiary smoke exposures or other carcinogenic substances from non-cigarette sources was not observed in this study.

Regardless of the small number of subjects, this study has proven the association between smoking habit and expression of the P16 protein of NPC tissue. With a larger number of samples, it will be interesting to observe as well how other carcinogens from environmental exposures may modify the association. Further, it is worth studying the mechanism(s) underlying the loss of P16 gene expression in NPC tissue. Additionally, closely observing the association between the loss of P16 gene expression and smoking habit with the prognosis of NPC patients is also worth pursuing, as it was previously observed that patients who smoked had lower P16 gene expression which lead to poorer prognosis among NPC patients (Hwang et al., 2002). 

In conclusion, in this study, a significant association between P16 and smoking status in Indonesian patients with NPC was observed. This study may raise the awareness of the mechanism(s) involved in the association of smoking habit and NPC, and further may be used to improve prevention, treatment and management of NPC.
